# Natal and Neonatal Teeth: An Overview of the Literature

**DOI:** 10.1155/2013/956269

**Published:** 2013-08-18

**Authors:** Shubhangi Mhaske, Monal B. Yuwanati, Ashok Mhaske, Raju Ragavendra, Kavitha Kamath, Swati Saawarn

**Affiliations:** ^1^Department of Oral Pathology and Microbiology, Peoples Dental Academy, Bhopal 462037, Madhya Pradesh, India; ^2^Department of Surgery, PCMS & RC, Bhopal 462037, Madhya Pradesh, India

## Abstract

The occurrence of natal and neonatal teeth is an uncommon anomaly, which for centuries has been associated with diverse superstitions among different ethnic groups. Natal teeth are more frequent than neonatal teeth, with the ratio being approximately 3 : 1. It must be considered that natal and neonatal teeth are conditions of fundamental importance not only for a dental surgeon but also for a paediatrician since their presence may lead to numerous complications. Early detection and treatment of these teeth are recommended because they may induce deformity or mutilation of tongue, dehydration, inadequate nutrients intake by the infant, and growth retardation, the pattern and time of eruption of teeth and its morphology. This paper presents a concise review of the literature about neonatal teeth.

## 1. Introduction

Natal teeth are teeth present at birth, and “neonatal teeth” are teeth erupted within the first month of life. Premature eruption of a tooth at the time of birth or too early is combined with many misconceptions. They are further accompanied by various difficulties, such as pain on suckling and refusal to feed, faced by the mother and the child due to the natal tooth/teeth. Some families are so superstitious that the afflicted child may be deprived of parental love. The family hopes that the offending teeth be removed as soon as possible.

Natal and neonatal teeth have been a subject of curiosity and study since the time it was first documented by Titus Livius, in 59 BC. Gaius Plinius Secundus (the Elder), in 23 BC, believed that a splendid future awaited male infants with natal teeth. In some countries, the child is considered to be monstrous and bearer of misfortune for example. As per Chinese tradition it is considered as a bad omen for girls [1].

## 2. Terminology and Synonyms

Dentitia praecox, dens connatalis, congenital teeth, fetal teeth, infancy teeth, predeciduous teeth, and precocious dentition are some of the terminologies used previously [1, 9, 12, 21, 65]. Lack of specificity and accuracy in description of the condition leads to subsequent discontinuity of these terms. The analogous terms of “natal” and “neonatal” teeth described by Massler and Savara are now most accepted [4]. These terms broadly describe the teeth that are erupted at birth or shortly thereafter. Although these terms only define the time of eruption and give no hint whether the tooth is a component of primary dentition or whether it is supernumerary, newer synonyms should be explored.

## 3. Proposed Classifications

The natal and neonatal teeth that do not confirm the criteria described for them and erupt within one to three and a half months are called *early infancy* teeth [66]. Few authors have tried to resolve the controversies in such cases. Spouge and Feasby [66] in 1966 classified, the natal & neonatal tooth on the basis of developmental stages whereas, Hebling et al. in 1997 classified according to the appearance of each natal tooth into the oral cavity [67, 68] (Table 1).

## 4. Incidence and Prevalence

Natal teeth are three times more common than neonatal teeth. The incidence of natal and neonatal teeth ranges from 1 : 2,000 to 1 : 3,500 [19, 23] (Table 2). The radiographic examination is essential to differentiate the premature eruption of a primary deciduous tooth from a supernumerary tooth [69]. Only 1% to 10% of natal and neonatal teeth are supernumerary. More than 90% of natal and neonatal teeth are prematurely erupted deciduous series of teeth, whereas less than 10% are supernumerary [17, 70, 71]. The supernumerary teeth should always be extracted, but the decision to extract a normal mature natal tooth should be done by taking into account local or general complications and parental opinion.

The most commonly occurs in the mandibular region of central incisors, followed by maxillary incisors, mandibular cuspids or molars, and maxillary cuspids or molars in descending order [23, 72] (Table 3). Natal or neonatal cuspids are extremely rare. 

There was no difference in prevalence between males and females. However, a predilection for female was cited by some authors. Anegundi et al. reported a 66% proportion for females against a 31% proportion for males [47]. 

## 5. Multifactorial Etiology 

Exact etiology for the premature eruption or for appearance of natal and neonatal teeth is not known. In the past, neonatal teeth were merely considered cysts of the dental lamina of the newborn [67]. Normally they appear corniform, white in colour, composed of compact keratin, and projected above the alveolar ridge [73]. 

It was also suggested that they occur due to inheritance as dominant autosomal trait. Endocrine disturbance resulting from pituitary, thyroid, and gonads also may be one of the key factors. Another hypothesis suggested is that excessive or increased resorption of overlying bone results in early eruption of the natal or neonatal teeth. Poor maternal health, endocrine disturbances, febrile episodes during pregnancy, and congenital syphilis are some of the contributing predisposing factors for the occurrence of natal and neonatal teeth suggested in the literature. However, according to Štamfelj et al. the occurrence of natal teeth associated with agenesis of their primary successors appears to be related to an accelerated or premature pattern of dental development rather than to superficial positioning of the tooth germs [74].

## 6. Environmental Predisposing Factors

Environmental factors could play an important role in eruption of neonatal teeth. Polychlorinated biphenyls (PCBs), polychlorinated dibenzo-*p*-dioxins (PCDDs), and dibenzofurans (PCDFs) seem to cause the eruption of natal teeth [74]. The only environmental factor that may be regarded as a causative factor of natal teeth is the toxic polyhalogenated aromatic hydrocarbons: PCBs, PCDDs, and PCDFs. They are among the most widespread environmental pollutants. They cross the placenta, and concentrations of PCDD/Fs in the adipose tissue of a newborn are correlated with those in mother's milk. The children with natal or neonatal teeth usually show other associated symptoms [38].

## 7. Syndromes Associated

Few syndromes are reported to be associated with natal teeth and neonatal teeth [8]. These syndromes include Ellis-Van Creveld (Chondroectodermal Dysplasia) [75], Pachyonychia Congenital (Jadassohn-Lewandowsky), Hallermann-Streiff (Oculomandibulodyscephaly with Hypotrichosis) [76], Rubinstein-Taybi, Steatocystoma Multiplex, Pierre-Robin, Cyclopia, Pallister-Hall, Short Rib-Polydactyly (type II), Wiedemann-Rautenstrauch (Neonatal Progeria), Cleft Lip and Palate, Pfeiffer, Ectodermal Dysplasia, Craniofacial Dysostosis, Multiple Steatocystoma, Sotos, Adrenogenital, Epidermolysis-Bullosa Simplex including Van der Woude, Down's Syndrome [77], and Walker-Warburg Syndromes [78].

## 8. Clinical Presentation

The natal teeth or neonatal teeth manifest usually with variable shape and size ranging from small, conical and may also resemble normal teeth. The appearance of these teeth is dependent on the degree of maturity, but most of the time they are loose, small, discoloured, and hypoplastic as in the cases presented here. They may show enamel hypoplasia/hypomineralization [79] and a small root formation suggestive of an immature nature. The majority of natal teeth may exhibit a brown-yellowish-/whitish-opaque colour [12]. 

They are attached to the oral mucosa in many instances as the root development is incomplete or defective. This leads to the mobility in teeth, with the risk of being swallowed or aspirated by the child. The mobility also may lead to degeneration of Hertwig's sheath which is responsible for the formation of root, thus resulting in further incomplete root development and stabilization. 

Increase in mobility could also cause changes in the radicular part of teeth such as cervical dentin, pulp cavity, and cementum as well. 

## 9. Histology

In a study of natal teeth, Hals [80] observed normal pulp tissue, except for the presence of inflammatory areas in some regions; moreover, Weil's basal layer and the cell-rich zone were absent [81]. Histologically, the thin layer of enamel or in extremely rare conditions absence of the enamel layer may be seen [77]. The enamel hypoplasia could be attributed to the disturbance/variation in amelogenesis process which was due to premature exposure of the tooth to the oral cavity. This may cause metaplastic alteration of the epithelium of the normally columnar enamel to a stratified squamous [80].

Dentino-enamal junction is not scalloped which similar to that found in deciduous teeth. Cervically dentin becomes atubular with spaces and enclosed cells [82]. Irregular dentinal tubules through the dentin along with calcospherites and predentin of various thicknesses could be present [33]. Atypical dentin was also observed in the natal/neonatal teeth which could have been the result due to the response to irritant stimulus from oral cavity. 

Developing teeth often had no cementum, and in those cases where acellular cementum could be observed it was thinner than normal.

Pulp canal and pulp chamber become wider in most of the cases. Vascularised pulps along with few inflammatory cells were also reported [83].

## 10. Ultrastructure Findings 

Jasmin and Clergeau-Guerithault [81] studied the surface topography of mandibular natal and neonatal incisors at the ultrastructural level using the scanning electron microscope (SEM). They observed that enamel of the teeth exhibited hypoplastic, depressed areas, and the incisal edge of natal tooth lacked enamel [81]. According to Uzamis et al., the thickness of enamel was around 280 microns compared to up to 1200 microns in normal teeth. This shows the retarded development of natal and neonatal teeth, because of incomplete mineralization at the time of birth [82].

In one of such extensive studies on natal and neonatal teeth, Masatomi et al. [55] reported that enamel has a normal prism structure and mineralization except in few cases where the prism structure was absent in the cervical part of the enamel. They also noticed that the cervical and apical dentin was tubular, and in developing teeth the dentin in these regions changed to an irregularly formed hard tissue of osteodentin character, in which enclosed cells could be observed. 

## 11. Complications 

A major complication from natal/neonatal teeth is ulceration on the ventral surface of the tongue caused by the tooth's sharp incisal edge. This condition is also known as Riga-Fede disease or syndrome [47]. Possibility of swallowing and aspiration which has already been described previously should also be one of the major concerns in complications. Other complications stated are injury to mother's breast and inconvenience during suckling. The consequences seen with the teeth include carious lesions, pulp polyp, or premature eruption of successor teeth.

## 12. Conclusion 

Natal and neonatal teeth diagnosis requires detailed case history accompanied by thorough clinical and radiographic examination of the infant. It is important to rule out by radiographic examination whether they are components of normal dentition or supernumerary to decide the treatment plan. The clinician should also assess the risk of haemorrhage due to the hypoprothrombinemia commonly present in newborns.

## Classification

- The appearance of each natal tooth in the oral cavity can be classified into four categories given as follows, as the teeth emerge in the oral cavity:
  1. shell-shaped crown poorly fixed to the alveolus by the gingival tissue and absence of a root;
  2. solid crown poorly fixed to the alveolus by the gingival tissue and little or no root;
  3. eruption of the incisal margin of the crown through the gingival tissues;
  4. edema of the gingival tissue with an unerupted but palpable tooth.
- Spoug and Feasby have suggested that, clinically, natal and neonatal teeth are further classified according to their degree of maturity.
  1. A mature natal or neonatal tooth is the one which is nearly or fully developed and has relatively good prognosis for maintenance.
  2. The term immature natal or neonatal teeth, on the other hand, implies a tooth with incomplete or substandard structure; it also implies a poor prognosis.
- If the degree of mobility is more than 2 mm, the natal teeth of category (1) or (2) usually need extraction.

## Figures and Tables

**Table 1:** Prevalence of neonatal and natal teeth in different populations and studies.

Authors	Prevalence	Number of children in the sample
Magitot, 1876 [2]	1 : 6000	17,578
Puech, 1876	1 : 30000	60,000
Ballantyne, 1896 [3]	1 : 6000	17,578
Massler and Savara, 1950 [4]	1 : 2000	6,000
Allwright, 1958 [5]	1 : 3408	6,817
Bodenhoff, 1959 [6]	1 : 3000	—
Wong, 1962 [7]	1 : 3000	—
Bodenhoff and Gorlin, 1963 [8]	1 : 3000	—
Mayhall, 1967 [9]	1 : 1125	90
Chow, 1980 [10]	1 : 2000 to 3500	—
Anderson, 1982 [11]	1 : 800	—
Kates et al., 1984 [12]	1 : 3667	7,155
Leung, 1986 [13]	1 : 3392	50,892
Bedi and Yan, 1990 [14]	1 : 1442	—
Rusmah, 1991 [15]	1 : 2325	9,600
To, 1991 [16]	1 : 1118	53,678
De Almeida and Gomide, 1996 [17]	1 : 21.6	1,019
Alaluusua et al.,* 2002 [18]	1 : 1000	34,457 (1997–2000)
El Khatib et al., 2005 [19]	1 : 3400	17000 (1984 and 2001)

*Exposed to toxin Finnish population-correlation with exposure to toxin and prevalence of neonatal teeth and natal teeth.

**Table 2:** Review of natal and neonatal teeth cases reported in the literature.

Sr. number	Author	Sex	Age	Number of teeth	Teeth position and number	Macroscopic features	Chief symptoms/complaint	Treatment
(1)	Ruschel H C et al., 2010 [20]	Male	14 days	1	Maxillary first molar right side	Calcified only at occlusal portion, no mobility	No complaint	Extraction

(2)	Deep et al., 2011 [21]	Female	22 days	1	Mandibular anterior	—	Ulceration over the ventral surface of tongue, no mobility, pain during sucking and feeding	Grinding and placement of composite over the teeth

(3)	Nandikonda, 2010 [22]	Female	10 days	2	Maxilla	Whitish opaque in color with a size similar to mandibular anterior region, crown portion was noted without any root structures	Cleft palate, causing feeding difficulty to the baby	Extraction

(4)	Dyment et al., 2005 [23]	Female	3 days	2	71 and 81	The teeth did not appear to be excessively mobile	Feeding without difficulty	Extraction

(5)	Shrestha, 2011 [24]	Female infant	12 days	2	Mandible, anterior teeth	Two teeth in the lower jaw since birth, whitish opaque in color and exhibiting grade III mobility	Mother complaining of pain on suckling and refusal to suck milk	Extraction

(6)	Chandra, 2011 [25]	Male	5 days	2	71, 81 mandibular anterior (natal)	Mobile, whitish opaque	Discomfort in feeding	Extraction
		Female	18 days	1	81 (neonatal)	Mobile, whitish opaque	Difficulty in breast feeding	Extraction
		Female	7 days	1	81 (natal)	Mobile, whitish opaque	Difficulty in breast feeding	Extraction

(7)		Female gender	6 hours	2	Primary central incisors (71 and 81) with	Root formation	Two injuries cyst (swelling small tissue soft/small nodule diameter 1 mm color translucent white) at the central region of the jaw	—
		Female	48 hours	2		—	Ulcer on the tongue Feeding difficulty	Extraction
	Gina et al., 2008 [26]	Male	9 days	1	Maxillary 51	—	(Small swelling of soft tissue/pellet 1 mm diameter small whitish translucent) at the central region of the mandible no uncomfortable and fed showed no complication (breastfeeding)	—
		Male	3 months	1	81 incisors	Appearance hypoplastic or hypomineralized (milky white *||*) Mobility grade type II, there was no associated injury	—	Periodic inspections and recommendations to the mother in relation to the hygiene and eating habits
		Female	5 months	1	71 mandibular incisors	—	—	Extraction

(8)	Marakoglu et al., 2004 [27]	Male	Stillborn	2	Two maxillary first incisors	—	—	—

(9)	Kaur et al., 2003 [28]	Male	4 months	1	—	—	Ulcer on ventral surface of tongue	Conservative t/t

(10)	Ndiokwelu et al., 2004 [29]	Female	4 days	1	Upper and lower teeth	—	Associated with Down syndrome	

(11)	Martinez, 2003 [30]	—	2 months	2	71, 81	Small root, hypoplastic enamel	Tooth mobility	Extraction

(12)	Rdos et al., 2011 [31]	Male	—	11	—	—	—	Prosthetic rehabilitation

(13)	Agostini et al., 2008 [32]	Male	4 months	2	71, 81	—	Nodular growth after exfoliation of teeth	—

(14)	Tomaki, et al., 2005 [33]	Male	27 days	1	81	Milky white and the other half yellowish brown with incomplete tooth crown-like hard tissue	Mobile mass with tooth-like hard tissue	Extraction

(15)	J. Kovac and D. Kovac, 2011 [34]	Female	5 weeks	2	71, 81	Hypoplastic	—	Extraction

(16)	Sibert and Porteous, 1974 [35]	Female (6)	3 days–6 months	8	71, 81	—	—	Extraction

(17)	Bartholin*		—	2 molars	—	—	—	—

(18)	Thomas*	—	—	8 incisors 1 molar	—	—	—	—

(19)	Bouchet*	—	—	2 mandibular incisors 1 mandibular molar	—	—	—	—

(20)	Jacobi*	—	—	1 max molar 1 mandibular molar 2 mandibular incisors	—	—	—	—

(21)	Kaufman*	—	—	4 mand molars 4 max molars	—	—	—	—

(22)	M lin*	—	—	2 molars				

(23)	Oriola*	—	—	2 mand molars	—	—	—	—

(24)	Allwright*	—	—	2 mand molars	—	—	—	—

(25)	Bodenhoff*	—	—	2 inciosrs 4 mand molars 4 max molars (1, 2nd)	—	—	—	—

(26)	Wong*	—	—	4 inciosrs 2 mand molars 2 max molars (1st)	—	—	—	—

(27)	Soni*	—	—	1 mand molar (1st)	—	—	—	—

(28)	Tay*	—	—	1 max molar (2nd)				

(29)	Bernick*	—	—	1 max molar (1st)	—	—	—	—

(30)	Ajagebe*	—	—	1 mand molar (2nd)	—	—	—	—

(31)	Anderson*	—	—	2 max molars (1st)	—	—	—	—

(32)	Ronk*	—	—	multiple incisors and molars	—	—	—	—

(33)	Primo et al., 1995 [36]	Female	6 months	2	71, 81	Two dental structures in which the incisor borders had no enamel and had exposed dentin. Mobility	The child cried during feeding, indicating pain and bleeding around two erupted teeth	Extraction

(34)	Basavanthappa et al., 2011 [37]	Female	15 days	1	81	Mobile, yellowish color, enamel hypoplasia	Difficulty in suckling	Extraction
		Female	19 days	1	81	Mobile, white color	Difficulty in suckling	Extraction
		Male	16 days	1	51	Mobile, white color	Cleft lip and palate	Extraction
		Female	14 days	1	81	Mobile, white color	Sublingual ulceration	Extraction
		Male	8 days	1	81	Mobile, white color	Difficulty in feeding	Extraction
		Female	18 days	1	71	Mobile, white color	Refusal to suck	Extraction
		Female	30 days	2	71, 81	Mobile, gingival inflammation	Refusal to suck, gingival inflammation	Extraction
		Male	25 days	1	81	Mobile, white color	Difficulty in feeding	Extraction
		Male	18 days	1	71	Mobile, white color	Sublingual ulceration	Extraction
		Female	17 days	1	71	Mobile, white color	Refusal to suck	Extraction
		Male	23 days	1	81	Mobile, white color	Refusal to suck	Extraction
		Female	21	1	71	Mobile, white color	Refusal to suck	Extraction
		Male	7 days	1	81	Mobile, yellowish color	Difficulty in suckling	Extraction
		Male	20 days	1	81	Mobile, white color	Difficulty in feeding	Extraction
		Female	21 days	1	71	Mobile, white color	Refusal to suck	Extraction

(35)	McDonald et al., 2004 [38]	Female	—	2	71, 81	Small, opaque, yellow, dysmorphic crowns	No difficulty to mother and child	Extraction (at age of 7 years)

(36)	Friend et al., 1991 [39]	Male	2 days	1 molar	54	A pale, globular tooth-like structure on the maxillary left alveolar ridge, rootless, mobile	—	Extraction

(37)	Kurian et al., 2007 [40]	Female	—	—	—	—	—	—

(38)	Taghi and Motamedi, 2009 [41]	Male	8 months	Mandibular incisor	—	—	Ulceration over ventral surface of tounge, difficulty in feeding	Grinding and placment of composite over the teeth

(39)	Sogi et al., 2011 [42]	Female	21 days	3 maxillary incisors	51, 61, 62	Mobile	Difficulty in feeding	Extraction

(40)	Venkatesh and Adhisivam, 2011 [43]	Female	3 months	2	71, 81	Yellowish with conical edges	Congenital hyperthyroidism, associated symptoms	Extraction

(41)	Roshan et al., 2009 [44]	—	2	2	51, 61	—	Hyper-IgE syndrome	—

(42)	Veena et al., 2011 [45]	Female	2 weeks	2	71, 81	—	Ellis van Creveld syndrome	Exfoliated

(43)	Rao et al., 2001 [46]	Female	25 days	2	71, 81	Whitish opaque in colour, mobility. The crown size was normal with no roots. Hypomineralized	Ulcer over ventral surface of tongue	Extraction

(44)	Anegundi et al., 2002 [47].	Female	30 days	1	71	Mobile, whitish opaque in colour	Localized inflammation, difficulty in feeding	Extracted
		Female	7 days	2	71, 81	Mobile, small yellowish brown in color	Difficulty in feeding	Extracted
		Male	10 days	2	74, 84	Mobile	Difficulty in feeding	Extracted
		Female	5 days	2	71, 81	Mobile, small, conical, yellowish brown, opaque teeth	Difficulty in feeding and refusal to suck	Extracted

(45)	Singh et al., 2004 [48]	Male	4 and 1/2 months	1	—	—	Pedunculated mass in relation to mandibular anterior tooth	Extraction

(46)	Ziai et al., 2005 [49]	Male	4 weeks	1 (premaxillary region-RT side)	—	—	Bilateral Cleft lip and palate, severe feeding difficulties and recurrent bleeding from movement of the loose tooth	Extraction
		—	5 days	1 (premaxillary region-RT side)	—	—	Difficulty in fabrication of device	Extraction

(47)	Hegde, 2005 [50]	Female	28 days	2	71, 81	Mobile, whitish in color	Ulceration over tongue, difficulty in sucking	Extraction

(48)	S. Sarkar and S. Sarkar, 2007 [51]	Male	3 months	1	54	Rootless	Difficulty in feeding	Extraction

(49)	Kumar et al., 2011 [52]	Female	3 months	3	54, 64, 65	Rootless	Early eruption and difficulty in feeding, crying	Exraction

(50)	Rao and Mathad, 2009 [53]	Female	2 days	2	71, 81	Whitish opaque in color, mobile	Difficulty in feeding and refusal to suck, crying	Extraction

(51)	Muraleekrishnan et al., 2011 [54]	Male	—	2	71, 81	—	—	Extraction

(52)	Masatomi et al., 1991 [55]	Male	18 months	Multiple	—	—	—	Extraction

(53)	Gonçalves et al., 1998 [56]	Male	1–6 days	12 (multiple)—8 in mandibular anterior region. 2 molars (max/mand)	—	Very little root formation	—	Extraction

(54)	Prabhakar et al., 2009 [57]	Female (twin)	1 month	1 1	71, 81	Mobility	Difficulty in feeding and suckling, and also the mother experienced discomfort feeding them	Extraction

(55)	Agostini et al., 2008 [58]	Male	4 months	2	71, 81	—	Nodular growth	Exfoliated

(56)	Dubois et al., 2010 [59]	Male	6 months	2	71, 81	—	Ulcer over ventral surface of tongue	Extraction

(57)	Eley et al., 2010 [60]	Female	11 months	2	71, 81	—	Ulceration over tip of tongue	Extraction

(58)	Samadi et al., 2011 [61]							

(59)	Slayton, 2000 [62]	Male	10 months	2	71, 81	—	Down syndrome	Smoothing of the incisal edge

(60)	Padmanabhan et al., 2010 [63]	Male	20 days	1	81		Large whitish lesion was observed on the undersurface of the tongue, difficulty in feeding	Neonatal tooth was smoothened to eliminate the sharp traumatizing edges followed by extraction teeth

*Data from 19 to 34 is adapted from [64].

**Table 3:** Details of our cases (total teeth).

Case number	Sex	Age	Teeth position and number	Macroscopic features	Chief symptoms/complaint	Treatment
1	Male	5 months	2 teeth (71 and 81) (neonatal)	Yellowish white. Partially formed root. Size as compared to normal deciduous central incisor, foramina	Neither the child nor the mother had any problem during breast feeding	Extraction
2	Male	3 days	2 teeth (71 and 81) (natal)	Yellowish white, smaller in size	Difficulty in feeding	Extraction
3	Male	2 months	2 teeth (71 and 81) (neonatal)	Yellowish white, open apical foramina, smaller in size	Pain and difficulty in feeding	Extraction
